# Dengue Serotype-Specific Differences in Clinical Manifestation, Laboratory Parameters and Risk of Severe Disease in Adults, Singapore

**DOI:** 10.4269/ajtmh.14-0628

**Published:** 2015-05-06

**Authors:** Chee-Fu Yung, Kim-Sung Lee, Tun-Linn Thein, Li-Kiang Tan, Victor C. Gan, Joshua G. X. Wong, David C. Lye, Lee-Ching Ng, Yee-Sin Leo

**Affiliations:** Communicable Disease Centre, Institute of Infectious Diseases and Epidemiology, Tan Tock Seng Hospital, Singapore; Environmental Health Institute, National Environmental Agency, Singapore; Yong Loo Lin School of Medicine, National University of Singapore, Singapore

## Abstract

Studies on serotype-specific features of dengue and disease severity on adults are limited. We prospectively recruited adult febrile patients without alternate diagnosis to dengue from April 2005 to December 2011. Outcomes were defined using both the World Health Organization (WHO) 1997 and 2009 criteria; Dengue hemorrhagic fever (DHF) and severe dengue (SD). Infecting serotype was identified in 469 dengue-confirmed patients comprising 22.0% dengue virus serotype 1 (DENV-1), 57.1% DENV-2, 17.1% DENV-3, and 3.8% DENV-4. Cases infected with DENV-1 were more likely to present with red eyes whereas presence of joint pain and lower platelet count was associated with DENV-2 cases. After adjusting for potential confounders, DENV-1 was associated with both DHF (adjusted Relative Risk [aRR] = 1.74) and SD (aRR = 2.1) whereas DENV-2 had a lower risk of DHF (aRR = 0.5). DENV-1 genotype 1 and DENV-2 cosmopolitan were the predominant genotypes identified. Infecting dengue serotype and possibly genotype may play an important role in disease severity among adult dengue patients in Singapore.

## Introduction

Dengue fever is the most prevalent arboviral infection worldwide, with up to 40% (2.5–3 billion people) of the world's population living in endemic regions. It is estimated that 50–80 million dengue infections occur each year, with 500,000 cases of dengue hemorrhagic fever (DHF), and at least 12,000–24,000 deaths, mainly among children under 15 years of age.^1^ However, the reemergence of dengue is increasingly associated with a shift in epidemiology to older cohorts with different clinical manifestations and severity compared with very young children.^2^ There is a need to better understand dengue in adult populations.

Dengue fever can be caused by any of four genetically related but antigenically distinct dengue virus (DENV) serotypes (DENV-1, DENV-2, DENV-3, and DENV-4).^2^ The dengue serotypes circulating all year round in Singapore are DENV-1, DENV-2, and DENV-3 with sporadic reports of DENV-4.^3^ The epidemiology of dengue in Singapore has evolved from a pediatric burden in the 1960s to a predominantly adult disease since the 1980s. Despite intensive vector control efforts, dengue remains endemic in Singapore with year round transmission and cyclical large outbreaks. Major outbreaks occurred in 2005 (326.5 cases per 100,000 population) which was dominated by DENV-1 followed by a DENV-2 outbreak in 2007 (192.3 cases per 100,000 population).^4^ There were 27 and 24 dengue deaths in 2005 and 2007, respectively. For the other years between 2005 and 2011, the number of dengue-reported deaths ranged from 6 to 10 per year. Although DENV-1 and DENV-2 are the main circulating serotypes, all four dengue virus are also detected.^5^ Infections with different serotypes may cause nearly identical clinical syndrome,^6^ but some differences in clinical manifestations have been reported, although conclusions are usually based on limited serotype comparisons and small sample sizes.^7^,^8^ The exception is a recent large cross-sectional study from the Americas comprising 1,716 children and adults.^9^ However, in view of differences in dominant genotypes circulating in the Americas compared with this part of world, there is currently no data to support extrapolation of these findings.

Clinically, dengue infection can range from mild dengue fever to severe plasma leakage with hemorrhagic manifestations. Multiple factors have been suggested to contribute to severe dengue (SD) such as secondary infections, age, viral load as well infecting serotype and genotype.^10^–^13^ Previous reports of dengue in children have suggested that infection with secondary DENV-2 is more likely to result in severe disease compared with other serotypes.^14^–^17^ In contrast, primary DENV-1 cases were more overt whereas primary DENV-2 and DENV-3 cases were usually silent.^18^,^19^ Furthermore, published phylogenetic data suggest that the predominant DENV-2 genotype in these studies is the Asian genotype rather than the Cosmopolitan genotype circulating in Singapore with as yet unknown impact on disease manifestation.^20^,^21^ Phylogenetic studies of the envelope protein gene of DENV have shown that even within DENV serotypes, there is extensive diversity resulting in various genotypes with varying epidemic potentials.^22^ Differences at serotype and molecular level are important not only for dengue endemic countries but for physicians in non-endemic countries in their management of febrile returned travelers infected with various dengue viruses circulating globally.

## Objective

In this study, we investigated dengue serotype-specific differences in clinical manifestations, hematological parameters, and plasma viral RNA level. We aimed to decipher dengue serotype-specific risk of severity in adult dengue in the context of available genotype data.

## Methods

### Ethical statement.

The National Healthcare Group Domain Specific Review Board approved the study (DSRB E/05/013, DSRB E/09/432) and written informed consent was obtained from all subjects. All data were anonymized.

### Patients.

The study was composed of dengue cohorts recruited from two service settings, primary care clinics and Communicable Disease Center, an infectious disease center that provides an outpatient walk-in service supporting Tan Tock Seng Hospital (TTSH), a university teaching hospital with 1,500 beds. Details of the primary care cohort were reported previously.^23^,^24^ Briefly, all adults presenting with an acute undifferentiated fever within 3 days of onset without alternate diagnosis to participating primary care settings from April 2005 to December 2011were recruited and included in this analysis. At the hospital, we recruited all acute undifferentiated febrile adults referred from emergency departments, primary care, other medical institutions as well as self-referrals regardless of duration of fever. Recruitment at the hospital started in January 2010 and ended in December 2011. Patients from the primary care recruitment site had three scheduled visits (fever days 1–3, fever days 4–7 and convalescent days 21–30) in which 93% completed the convalescent visit. Patients from the hospital recruitment site were followed up daily during the acute phase until defervescence and rising platelet count with a final visit during convalescent days 21–30. During the visits patients were prospectively reviewed for detailed symptoms and signs, especially clinical evaluation for pleural effusion and ascites; serial blood evaluation including full blood count, hematocrit, and protein levels was carried out according to the study schedule. Decubitus chest x-ray and ultrasound were not routinely performed, but only when there was clinical suspicion. For the cases that required inpatient care from both cohorts, detailed daily hospital data were collected from review of the case notes until discharged.

### Laboratory methods.

Dengue was confirmed and serotype determined by real-time reverse transcription-polymerase chain reaction (RT-PCR) using an in-house protocol using the samples from the first day of presentation.^25^ Viral RNA levels were expressed as pfu (plaque-forming units)/mL of plasma using internally validated conversions from threshold cycle (*C*_t_) values of RT-PCR. RT-PCR negative cases that tested positive for non-structural protein 1 (NS1) antigen using Bio-Rad (Hercules, CA) Dengue NS1 Ag strip were also defined as confirmed dengue cases.^26^ Sequencing of the envelope protein gene of DENV had previously been described.^3^,^27^ Phylogenetic analysis of DENV sequences was conducted by using the maximum-likelihood method as implemented in PAUP* software (Sunderland, MA), version 4.0b10 (8), and compared with sequence data obtained from GenBank. Serology testing was carried out using: Platelia^™^ NS1 ELISA (Bio-Rad Laboratories, Marnes-la-Coquette, France), Panbio^®^ Dengue IgG Indirect, IgG Capture, and IgM Capture ELISAs (Alere Inc., Waltham, MA). Confirmed cases with samples that are negative by IgG Indirect or Capture assay during the acute phase were classified as primary cases. An acute sample positive by IgG Capture or an early acute sample positive by IgG Indirect defined secondary infection status in confirmed dengue cases.

### Outcome variables.

The clinical manifestations included in the analysis were headache, drowsiness, eye pain, muscle pain, joint pain, rash, bleeding, anorexia, nausea, vomiting, red eyes (conjunctival injection), and abdominal pain. The World Health Organization (WHO) 2009 warning signs of lethargy/drowsiness, severe abdominal pain, and mucosal bleeding were assessed as one variable defined as patients who fulfilled any of the three warning signs (WS). Data on the other WS were not collected for the whole cohort and hence were not included in the analysis. Plasma viral RNA level, temperature, systolic blood pressure, pulse rate, hematocrit, platelet, and leukocyte count at the first day of presentation were analyzed. Both DHF and SD were classified according to the WHO 1997 and 2009 criteria; data from entire clinical course were used to assess disease severity outcomes. All four of the following criteria must be present to fulfill the case definition of DHF, namely: fever or history of fever, hemorrhagic tendencies, thrombocytopenia, and evidence of plasma leakage.^28^ SD was defined by one or more of the following: severe plasma leakage (shock, fluid accumulation with respiratory distress), severe bleeding, or severe organ impairment.^29^

### Data analysis.

For descriptive analyses, number and percentage were used for categorical variables; median and range were used for continuous variables. The χ^2^ test was used to compare univariate categorical data whereas Fisher's exact test was used if expected cell sizes < 5. Modified Poisson regression was carried out for binary outcomes whereas ordinal logistic regression was used for ordinal categorical data.^30^ Adjustment was done for clinically relevant potential confounders: age, gender, year of infection, recruitment site, fever day, and primary/secondary infection status unless stated otherwise. All analysis was done using R 15.0 and SAS 9.2 (SAS Institute, Cary, NC).

## Results

### Demographics.

Between April 2005 and December 2011, a total of 3,468 patients were enrolled into our study cohorts. Dengue was confirmed in 617 (18.2%) patients. Serotype information was available for 469 (76%) of dengue-confirmed patients comprising 103 (22.0%) DENV-1, 268 (57.1%) DENV-2, 80 (17.1%) DENV-3, and 18 (3.8%) DENV-4. Dual infection with different serotypes was not detected in any cases. Cases where infecting serotype could not be determined comprised mainly younger individuals (median age 31 years), late presenters at the hospital rather than primary care with a median of fever day 7 and were less likely to be admitted. There were no significant differences in terms of gender, ethnicity, and proportion with secondary infection. DENV-4 cases were not included in subsequent statistical analysis in view of the low numbers. Notably 55.7% of the cohort, 53.1% of DENV-1, 60.8% of DENV-2, and 46.3% of DENV-3 were secondary infections (Table 1). In all, 64.1% (66) of DENV-1, 41.8% (112) of DENV-2, and 46.3% (37) of DENV-3 cases required hospital admission.

Univariate analysis showed that there were significant differences between patients for the three predominant dengue serotypes in terms of gender, primary/secondary infection status, recruitment site, and fever day at presentation (Table 1). DENV-2 had the highest proportion of males (74.3%) compared with DENV-1 (68.5%) and DENV-3 (58.8%). Secondary cases were more common among DENV-2 cases (60.8%) followed by DENV-1 (53.1%) and DENV-3 (46.3%). Eighty-five percent (85%) of DENV-3 and 83.3% of DENV-1 cases were recruited from primary care in contrast to only 45.9% of DENV-2 cases due to the earlier start date of the primary care study and the evolving epidemiology of dengue in Singapore during the study period described earlier. Consequently, cases of DENV-3 and DENV-1 were more likely to present on average 1 day earlier following onset of fever (*P* < 0.001).

### Clinical manifestation and hematological parameters.

After adjusting for age, gender, year of infection, recruitment site, fever day, and primary/secondary infection status, cases infected with DENV-1 were more likely to have red eyes (relative risk [RR] = 1.61, 95% confidence interval [CI] = 1.13–2.29) in contrast to DENV-2 for which the sign was less likely to be observed (RR = 0.74, 95% CI = 0.60–0.92). Instead, DENV-2 cases were more likely to present with joint pain (RR = 1.19, 95% CI = 1.04–1.35). There were no differences in the fulfillment of WHO 2009 warning signs of drowsiness, abdominal pain, and mucosal bleeding between the three serotypes (Table 2). There were significant serotype specific differences for platelet count with DENV-2 cases having the lowest platelet count with a median of 114 × 10^9^/L compared with DENV-1 (128 × 10^9^/L) and DENV-3 (141.5 × 10^9^/L) cases (*P* < 0.01) (Table 3). No differences were found between the serotypes in terms of temperature, systolic blood pressure, pulse rate, hematocrit value, and leukocyte count at presentation.

### Disease severity.

Crude analysis showed that there was no difference in disease severity between DENV-1, DENV-2, and DENV-3 when using WHO 1997 criteria in terms of DHF. However, when using WHO 2009 criteria, DENV-1 was found to have a higher risk of SD (crude RR = 2.5, 95% CI = 1.8–6.0). After adjusting for age, gender, year of infection, recruitment site, fever day, and primary/secondary infection status, the higher risk of SD for DENV-1 remained statistically significant and DENV-1 demonstrated a statistically significant higher risk of DHF as well (Table 4). In contrast, DENV-2 cases had a lower risk of DHF (adjusted RR = 0.5, 95% CI = 0.35–0.75).

### Plasma viral RNA level.

**Figure 1.**
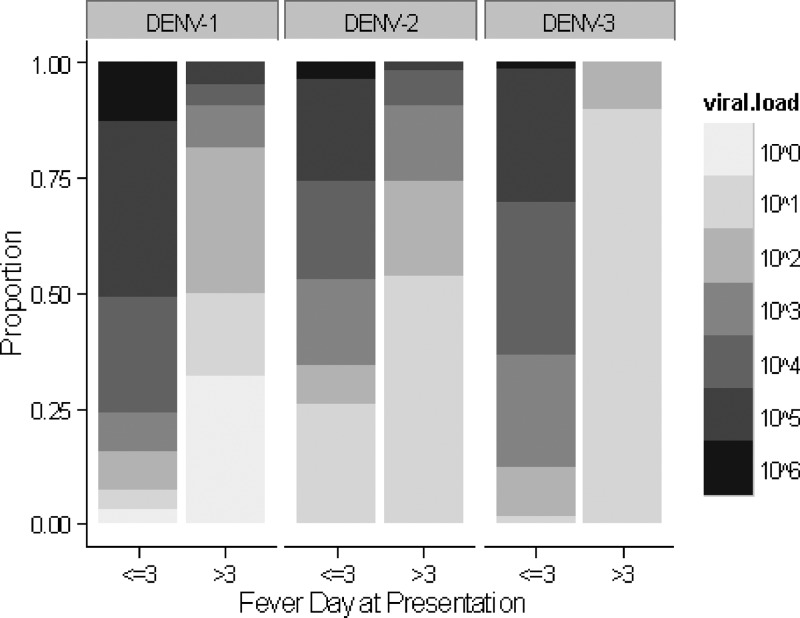
Distribution of plasma viral RNA level (pfu/mL) at the first day of presentation by dengue serotypes and fever day at presentation (≤ 3 days and > 3 days). Pfu = plaque-forming units.

In Figure 1, a trend for higher viral RNA level in DENV-1 cases was observed. After adjusting for fever day at presentation, primary/secondary infection status, and DHF/DF in an ordinal logistic regression analysis, DENV-1 viral RNA level was almost two times higher (adjusted odds ratio [OR] = 1.65, 95% CI = 1.21–2.08) compared with DENV-2 and DENV-3 cases. In contrast, viral RNA level in DENV-2 cases were significantly lower by nearly half compared with the other serotypes (adjusted OR = 0.59, 95% CI = 0.20–0.98).

### Genotype.

E-envelope sequencing information was available for 272 (58%) of the patients in our cohort. The main genotypes identified for each specific serotype were DENV-1 genotype 1 (95%, *N* = 66/70), DENV-2 cosmopolitan (100%, *N* = 138), DENV-3 genotype 3 (82%, *N* = 44/54), and DENV-4 genotype 2 (100%, *N* = 10). In terms of outcome severity, 63% (*N* = 12/19) of DENV-1 DHF cases were from genotype 1, 87% (*N* = 45/52) of DENV-2 DHF cases were from the cosmopolitan genotype and 46% (*N* = 5/11) of DENV-3 DHF cases were from genotype 1.

## Discussion

The risk of developing DHF and SD in DENV-1 patients was apparently higher compared with patients with DENV-2 or DENV-3. We found that DENV-1 cases were more likely to present with red eyes that contrasted with DENV-2 cases for which the sign was less likely. Instead, DENV-2 cases were more likely to develop joint pains as well as have low platelet count at presentation. Viral RNA levels were found to be significantly higher in DENV-1 cases and lower in DENV-2 cases which may provide a biological basis for the differences identified in our analysis. Phylogenetic analysis suggested that these differences may be attributable to DENV-1 and DENV-2 viruses from genotype 1 and cosmopolitan, respectively.

Our study cohort which was composed of more male dengue cases is not unusual in Singapore where previous data consistently showed male gender being at higher risk of dengue infections.^31^ The higher proportion of secondary infection in DENV-2 followed by DENV-1 and DENV-3 in our cohort reflected the epidemiology of dengue in Singapore where the DENV-2 is the main serotype since 2007 after a major outbreak of DENV-1 in 2005.^4^ Most DENV-1 cases were recruited from primary care since enrollment at the site started in 2005 just before a national DENV-1 outbreak. Similarly, since cases of DENV-1 as well as DENV-3 were mostly recruited from a primary care setting, they tended to present earlier resulting in fever day at presentation 1 day earlier than DENV-2 cases, about half of whom were recruited from the tertiary care setting.

Excluding the large study by Halsey and others in the Americas, previous studies in Asia investigating adult dengue serotype-specific clinical manifestations which included multiple dengue serotypes for comparison in the analysis could not identify any significant differences.^32^–^34^ However, these studies were small with the largest sample size comprising 126 dengue cases. We found red eyes as a clinical manifestation more likely to be associated with DENV-1 infection in contrast to DENV-2 infection. Halsey and others did not include red eyes as a clinical sign but identified retro-orbital pain as being more common in their DENV-1 cases but less so in DENV- 3 cases. We found joint pain to be more likely in DENV-2 cases whereas Halsey and others found in their study the same symptom was less common in DENV-1 cases. Our cohort had a larger number of DENV-2 cases whereas Halsey's cohort had more power for the other serotypes. It is interesting to note the corroboration of serotype specific clinical manifestations from both studies. Further studies with larger samples from all serotypes will be needed to confirm these serotype specific clinical manifestations.

A study in Hong Kong found that DENV-3 had lower minimum lymphocyte count compared with other serotypes.^32^ Although the median leukocyte count for DENV-3 cases in our study was the lowest, this was not statistically significant. The only hematological parameter that was significantly different between serotypes was platelet count with DENV-2 cases having the lowest median platelet count. It is unclear what these possible parameter differences represented in view of the dynamic nature of dengue disease. The small differences in platelet count level between dengue serotypes could also be attributable to measurement variations. However, we did find a strong correlation between DENV-1 with severe outcome using either WHO 2009 or WHO 1997 classification of SD or DHF, respectively, compared with DENV-2 and DENV-3 after adjusting for clinically relevant confounders. In contrast, DENV-2 infection was found to be the least likely to progress to DHF despite cases presenting with lower platelet counts. The higher plasma viral RNA level found in cases of DENV-1 and lower level in DENV-2 cases provided a possible biological basis for these severity differences. However, the assumption that dengue viral load is an important predictor of disease severity remains uncertain with conflicting study findings.^17^,^35^

Previous studies demonstrated that Asian DENV-2 and DENV-3 genotypes originating from southeast Asia correlated with increased incidences of DHF and dengue shock syndrome in the Americas, South Pacific, and Sri Lanka.^36^–^38^ Ours is the first study to investigate the clinical pathogenesis of adult DENV-2 cosmopolitan genotype which is the predominant strain in Singapore, Malaysia, and Borneo.^39^–^41^ Since up to 87% of the DHF cases in DENV-2 cases were from the cosmopolitan genotype, it is possible that the reduced severity of DENV-2 identified in our study may be attributed to DENV-2 cosmopolitan genotype. This finding lends support to the hypothesis that virus strain and small genotypic changes may be important modifying factors for severity and hence phylogenetic analysis is important when interpreting serotype-specific data. Full length sequencing of DENV-2 Asian genotype compared with DENV-2 American genotype suggested that variations at position 390 in the E protein and in the 5′ and 3′ untranslated regions (UTR) may be responsible for the difference in severity through increased replication in humans and mosquitoes.^38^,^42^,^43^ Future work to sequence the DENV-2 cosmopolitan genotype will provide an interesting opportunity to investigate this hypothesis further as well as identifying variations that may be correlated with less severe disease manifestations. Such information will allow public health authorities to advance virus surveillance from serotype monitoring to identifying markers of genotypic fitness.

A limitation of this study is the absence of serotype information for about 24% of confirmed dengue cases recruited. We were unable to include DENV-4 in our analysis due to small numbers although it is usually regarded as the most clinically mild serotype.^16^ We did not have information on sequence of infection by different dengue serotypes and therefore we cannot compare with previous studies that have examined this.^18^ This may have an important impact on our findings. Decubitus chest x-ray and ultrasound were performed on clinicians' decision, thus the detection of clinical fluid accumulation may not be standardized. We used clinical, hematological and viral load data at the first day of presentation for medical care which may not reflect the complete picture of the disease course. However, using only information available at the day of presentation provided a realistic clinical management scenario. This would not have affected our severity analysis that was based on information over the whole course of the illness. In view of the different start times and sites for recruitment as well as changing dengue epidemiology, we included adjustment for recruitment site and year of infection in the multivariable analysis. We also controlled for clinically important confounders of age, gender, fever day at presentation, and primary/secondary infection status when necessary but we cannot exclude the possibility of unknown confounders resulting in bias in our result. Although genotype data were not available for all serotyped cases to analyze the direct relationship between dengue genotypes with outcome, only 7 of the total 52 DENV-2 DHF cases had unknown genotype while the rest were identified as DENV-2 cosmopolitan. Furthermore, ongoing molecular surveillance data confirmed the dominance of DENV-2 cosmopolitan in Singapore with rare occasional cases of DENV-2 Asian genotypes identified.^41^

## Conclusions

We demonstrated that DENV-1 infection may be more severe compared with DENV-2 infection. Findings from our prospective adult dengue cohort found that DENV-1 cases were more likely to present with red eyes whereas absence of red eyes but presence of joint pain and lower platelet count was associated with DENV-2 cases. The differences in severity may be attributable to variations in plasma viral RNA levels between serotypes. At a molecular level, our findings may be associated with DENV-1 genotype 1 and DENV-2 cosmopolitan genotype.

## Figures and Tables

**Table 1:** Patient demographics

Variables	DENV-1 (*N* = 103)		DENV-2 (*N* = 268)		DENV-3 (*N* = 80)		*P* value
	*N*	%	*N*	%	*N*	%	
Male	64	66.7	199	74.3	47	58.8	**< 0.01**
Secondary infection	51	53.1	163	60.8	37	46.3	**0.02**
Year of infection							**< 0.01**
2005	66	68.8	5	1.9	63	78.8	
2006	3	3.1	1	0.4	0	0	
2007	0	0	46	17.2	0	0	
2008	6	6.3	17	6.3	0	0	
2009	0	0	8	3	3	3.8	
2010	19	19.8	94	35.1	8	10	
2011	9	9.4	97	36.2	6	7.5	
Chinese ethnicity	79	82.3	192	71.6	66	82.5	0.13
Recruited from primary care center	80	83.3	123	45.9	68	85	**< 0.01**
Age, median (range)	35 (19–74)		37 (18–77)		39 (19–87)		0.13
Fever day at presentation, median (range)	2 (1–7)		3 (1–9)		2 (1–9)		**< 0.01**

DENV-1 = dengue virus (DENV) serotype 1.

Values in bold are statistically significant.

**Table 2:** Clinical manifestations by dengue serotypes and multivariable model comparing each dengue serotype to the other two serotypes

Variables	DENV-1 (*N* = 103)			DENV-2 (*N* = 268)			DENV-3 (*N* = 80)		
	*N*	%	RR* (95% CI)	*N*	%	RR* (95% CI)	*N*	%	RR* (95% CI)
Headache	87	84	1.26 (0.78–2.02)	214	80	1.01 (0.87–1.19)	59	74	0.70 (0.48–1.02)
Eye pain	24	23	0.83 (0.58–1.17)	68	25	1.13 (0.99–1.30)	17	21	0.82 (0.53–1.26)
Muscle pain	71	68	0.90 (0.65–1.25)	192	71	0.91 (0.79–1.05)	59	74	1.28 (0.84–1.94)
Joint pain	61	60	0.95 (0.68–1.32)	163	61	**1.19 (1.04**–**1.35)**	42	53	0.78 (0.55–1.11)
Rash	20	19	1.16 (0.73–1.84)	65	24	0.88 (0.74–1.03)	15	19	1.25 (0.79–1.99)
Anorexia	91	88	1.16 (0.71–1.89)	228	85	1.08 (0.91–1.31)	66	82	0.76 (0.48–1.19)
Nausea	60	58	1.14 (0.83–1.57)	172	64	1.06 (0.92–1.22)	38	47	0.80 (0.55–1.15)
Vomiting	22	21	1.09 (0.75–1.59)	56	20	0.97 (0.83–1.13)	16	20	1.0 (0.65–1.54)
Red eyes	34	33	**1.61 (1.13**–**2.29)**	38	14	**0.74 (0.60**–**0.92)**	20	25	0.88 (0.57–1.35)
Drowsiness	67	65	1.11 (0.79–1.49)	161	60	0.98 (0.86–1.11)	47	59	0.88 (0.62–1.25)
Bleeding	12	11	0.96 (0.57–1.63)	39	14	0.92 (0.77–1.09)	11	14	1.46 (0.84–2.46)
Abdominal pain	4	4	0.58 (0.22–1.53)	47	17	1.02 (0.90–1.16)	5	6	1.61 (0.57–4.48)
Warning signs†	74	72	1.21 (0.84–1.73)	186	69	0.99 (0.87–1.14)	49	61	0.82 (0.58–1.17)

CI = confidence interval; DENV-1 = dengue virus (DENV) serotype 1; RR = relative risk.

Values in bold are statistically significant.

* Modified Poisson regression adjusted for age, gender, primary/secondary infection, recruitment site, year of infection, and fever day at presentation.

† Drowsiness, bleeding or abdominal pain.

**Table 3:** Dengue serotype-specific differences in vital signs and hematological parameters

Variables	DENV-1 (*N* = 103)		DENV-2 (*N* = 268)		DENV-3 (*N* = 80)	
	Median (range)	*P* value*	Median (range)	*P* value*	Median (range)	*P* value*
Temperature (°C)	38.3 (36.1–40.6)	0.050	37.9 (35.9–40.3)	0.30	38 (36.2- 40.3)	0.15
Systolic blood pressure (mmHg)	112 (76–146)	0.11	118 (74–171)	0.70	116.5 (78–149)	0.46
Pulse/minute	91 (60–133)	0.30	83 (45–144)	0.90	87.5 (59–136)	0.42
Hematocrit (%)	43.8 (26.574.5)	0.34	44.1 (15.1–71.9)	0.26	43.3 (26.8–58.1)	0.90
Platelet count (10^9^/L)	**128 (8**–**383)**	**0.046**	**114 (8**–**450)**	**< 0.01**	141.5 (16–388)	0.92
Total leukocyte count (10^9^/L)	3.2 (1–15.3)	0.55	3 (0.5–27.1)	0.88	3.2 (1.1–9.8)	0.15

DENV-1 = dengue virus (DENV) serotype 1.

Values in bold are statistically significant.

* Modified Poisson regression comparing one serotype to the other two serotypes adjusted for age, gender, primary/secondary infection, recruitment site, year of infection, and fever day at presentation.

**Table 4:** Dengue virus serotypes and disease severity

Serotype	*N*	DHF			SD		
		*N* (%)	RR (95% CI)	aRR* (95% CI)	*N* (%)	RR (95% CI)	aRR* (95% CI)
DENV-1	103	19 (18)	1.01 (0.52)	**1.74 (1.1**–**2.7)**	13 (13)	**2.5 (1.8**–**6)**	**2.1 (1.1**–**4)**
DENV-2	268	52 (19)	1.18 (0.72–2.6)	**0.5 (0.35**– **0.75)**	13 (5)	0.5 (0.26–1.05)	0.85 (0.3–2)
DENV-3	80	11 (13)	0.7 (0.11–0.9)	1.4 (0.8–2.3)	4 (5)	0.7 (0.25–2)	0.4 (0.12–1.25)

DHF = dengue hemorrhagic fever; SD = severe dengue.

Values in bold are statistically significant.

* Modified Poisson regression adjusted for age, gender, primary/secondary infection, recruitment site, and year of infection.
